# Structural and Functional Effect of an Oscillating Electric Field on the Dopamine-D3 Receptor: A Molecular Dynamics Simulation Study

**DOI:** 10.1371/journal.pone.0166412

**Published:** 2016-11-10

**Authors:** Zohreh Fallah, Yousef Jamali, Hashem Rafii-Tabar

**Affiliations:** 1 School of Nano-Science, Institute for Research in Fundamental Sciences (IPM), Tehran, Iran; 2 Department of Mathematics, Tarbiat Modarres University, Tehran, Iran; 3 Department of Medical Physics and Biomedical Engineering, Faculty of Medicine, Shahid Beheshti University of Medical Sciences, Evin, Tehran, Iran; Indian Institute of Technology Kanpur, INDIA

## Abstract

Dopamine as a neurotransmitter plays a critical role in the functioning of the central nervous system. The structure of D3 receptor as a member of class A G-protein coupled receptors (GPCRs) has been reported. We used MD simulation to investigate the effect of an oscillating electric field, with frequencies in the range 0.6–800 GHz applied along the z-direction, on the dopamine-D3R complex. The simulations showed that at some frequencies, the application of an external oscillating electric field along the z-direction has a considerable effect on the dopamine-D3R. However, there is no enough evidence for prediction of changes in specific frequency, implying that there is no order in changes. Computing the correlation coefficient parameter showed that increasing the field frequency can weaken the interaction between dopamine and D3R and may decrease the Arg128{3.50}-Glu324{6.30} distance. Because of high stability of α helices along the z-direction, applying an oscillating electric field in this direction with an amplitude 10-time higher did not have a considerable effect. However, applying the oscillating field at the frequency of 0.6 GHz along other directions, such as X-Y and Y-Z planes, could change the energy between the dopamine and the D3R, and the number of internal hydrogen bonds of the protein. This can be due to the effect of the direction of the electric field vis-à-vis the ligands orientation and the interaction of the oscillating electric field with the dipole moment of the protein.

## Introduction

G protein-coupled receptors (GPCRs) are one of the largest superfamily of membrane proteins in human body. These membrane receptors, by interacting with G-proteins, play an important role in different signal transduction pathways. External stimuli, such as light, smell, and taste and the binding of specific ligands and hormones to the extracellular regions of these receptors, lead to conformational changes of receptors and then result in the activation of the G-protein.[1,2] Due to biological and pharmaceutical importance of GPCRs in several diseases such as migraine, Parkinson’s, schizophrenia etc, considerable experimental efforts have been spent to understand the structure and function of GPCRs so as to design efficient drugs.[3–8] The crystal structures of different GPCRs in complex with agonists and antagonists have been determined such as the human β_2_-adrenergic receptor (β_2_AR) [PDB: 2RH1], the rhodopsin [PDB: 3DQB], the human A2A-adenosine receptor [PDB: 3EML], the M_2_ and M_3_ muscarinic receptors [PDBs: 3UON and 4DAJ], the histamine H_1_ receptor [PDB: 3RZE] and the Dopamine D_3_R [PDB: 3PBL].[9–15]

Dopamine is an important neurotransmitter in the central nervous system that plays a critical role in movement, cognition, and emotion. Imbalance of the dopaminergic system leads to neuropsychiatric disorders such as Parkinson’s disease, Huntington’s disease, schizophrenia, Tourette’s syndrome, and drug abuse.[16–18] All dopamine receptors belong to the GPCR superfamily which has been classified under two subfamilies. The D_1_-like receptors consist of D_1_ and D_5_ receptors that couple to stimulatory G_s_ protein, activating adenylcyclase, and D_2_-like receptors, including D_2_, D_3_ and D_4_ receptors, that couple to inhibitory G_i/o_ proteins and inhibit adenylcyclase.[19–22] The atomic structure of D3R was resolved by x-ray crystallography at 2.9 Å of resolution [PDB entry code 3PBL].[15] There is a high degree of sequence identity (78%) within the transmembrane (TM) helices between D2R and D3R, creating a challenge to develop D3R-selective drugs with physicochemical properties.[23–26]

D3R, like other GPCRs consists of the seven-TM bundle of α helices connected by loop regions, but the ICL2 forms a 2.5-turn α helix that runs parallel to the membrane. Subtle differences in the orientation of α helices and differences in the intracellular and extracellular parts give the receptor their unique biochemical properties. For example the extracellular tips of helices 3 and 5 are 3.5 Å closer to each other in D3R than those in β_2_AR.[15] A common property thought to be important in many class A GPCRs is the ionic lock, a salt bridge between the charged Arg128^{3.50}^ and Glu324^{6.30}^ which is an important parameter to stabilize the protein in inactive conformation.[27–33] Receptor activation is thought to involve conformational changes in the form of both helical movements, primarily a tilt of helix 6 as formulated in the “global toggle switch” model,[34–36] as well as the rotameric alteration of key side-chains, referred to as “activation micro-switches”.[37–42]

Time-varying electric fields can affect biological systems for example in such phenomenon as electroporation. An external alternating current (ac) field increases the permeability of the cell membrane in the course of several biological processes.[43,44] Exposure to GHz frequency in communication technology and various devices may affect the proteins, such as receptors, in many different ways, for example via the interactions of protein dipole moments with the external oscillating electric field. In general, charged and polar residues can directly interact with an oscillating electric field, and this may lead to some perturbation in the proteins.

With access to the atomic structure of receptors, molecular simulations can be employed to investigate the receptors at atomistic level. These simulations can provide access to the dynamics of microscopic structure of a GPCR in the membrane and its interaction with the surrounding solvent and the ions at the atomistic level. Different computational simulations have been performed to investigate the dynamics of GPCRs.[44–48] For example, recently distinct conformations of the ionic lock (broken and salt-bridged) of the inactive antagonist bound β_2_AR were revealed by conventional molecular dynamics (cMD) simulation.[49] The effects of external electric fields on proteins, like potassium channel and pump, have been investigated by molecular simulations and different continuum-based modeling.[50,51]

Considering the importance of dopamine and D3R as a protein with the available atomistic structure and because of the presence of the static and oscillating electric fields in the environment, the response of D3R and dopamine to external electric fields form important issues. Here, we are not looking for the main shift of substates within GPCRs which is in the order of hundreds of nanoseconds. In this paper, we are interested in understanding the molecular mechanism underlying the interaction of an external electric field with the D3R. We employ MD simulation to investigate this effect on the structure of D3R in complex with the dopamine in the limited simulation time. The organization of the paper is as follows. Following the Introduction in Section I, we consider the simulation method and the data employed in this paper in Section II. Section III contains the results of our simulations pertinent to the cases when an oscillating field, at various frequencies, was applied along the z-direction only, when a high amplitude field was applied, again in the z-direction only, and finally when the oscillating field was applied along other directions. Section IV summarizes our work.

## Computational method

The 2.89 Å resolution x-ray crystal structure of D3R, under the name PDB ID 3PBL, was taken from the Protein Data Bank (PDB).[15] Since the extracellular end of helix I was not resolved in the D3R crystal structure, in order to fit the protein in the lipid bilayer [15] 3 residues (29–30) and also missing atoms were added to the protein by Swiss-Pdb Viewer software.[52]

**Fig 1:**
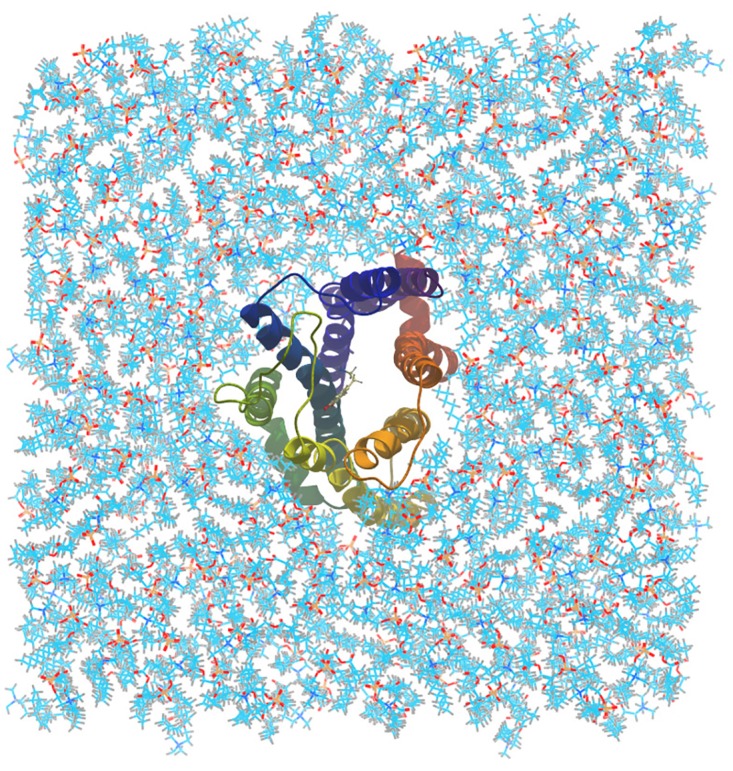
D3R-dopamine complex embedded in POPC membrane.

An appropriate orientation of the protein in the membrane was found by Server-Orientations of Proteins in Membranes (OPM) database.[53] The 1-palmitoyl-2-oleoylsn-glycero-3- phosphatidylcholine (POPC) using the Membrane plugin distributed in VMD [54] was used as a lipid bilayer which was equilibrated for at least 50 ns at T = 310 K and 1 atm within an NPT ensemble with anisotropic pressure coupling. D3R was oriented along the z-direction into the lipid bilayer by g_membed in GROMACS 4.5.4 software (Fig 1).[55] All-atom molecular simulation was performed by GROMACS 4.5.4 with the parameters of the charmm27 force field (FF) for protein and lipid. SwissParam was used to create the topology and parameter files needed in CHARMM for dopamine [56]. The simulation box had the dimensions 95Å×95Å×120Å, large enough to remove any artifacts arising from the imposition of the periodic boundary conditions (PBC).[57] The TIP3P water model was chosen and Cl^−^and Na^+^ ions were added in 0.15 M concentration (natural ion concentration) to keep the system neutral. The simulation temperature was set at T = 310 K and the time step set at 2 fs. The particle-mesh Ewald method was used to compute the electrostatic interactions. The system consisted of a protein, 228 lipid molecules, 19074 water molecules, 91 chlorine ions and 83 sodium ions.

Following minimization and equilibration of the protein in the membrane for 8 ns, the dock program CDocker [58] was applied to build the dopamine D3R complex. The radius of 20 Å was considered on docking to cover the binding pocket appropriately. The best binding energy for the complex was chosen which was in accordance with other binding results.[59] It was equilibrated for 4 ns within the constant-NPT ensemble using the Nose–Hoover thermostat and Parrinello–Rahman barostat for scaling the temperature and the pressure, respectively. The system in F = 0 and different frequencies was simulated 4 times independently, each for 20 ns, to obtain the best results. The system was exposed to an oscillating electric field of the form E(t) = E_0_ cos(wt) by GROMACS 4.5.4 [55], with different logarithmic frequencies such as 0.6, 0.8, 1, 1.5, 2, 2.1, 2.9, 3, 3.1, 4, 5, 6, 7, 8, 9, 10, 12, 15, 18, 20, 21, 22, 50, 120, 300 and 800 GHz applied in the z-direction. The amplitude of the field was chosen with the rms intensity of 0.065 V/nm,[60] and in some simulations, higher amplitude was also applied.

Since the polarizability of atoms is not considered in molecular dynamics simulation, main dielectric response of atoms to the applied electric field occurs through molecular reorientation, i.e. the waters, ions and charged and polar residues in protein. Besides, at high frequencies, molecular reorientation times would be longer than field period, and then therefore have negligible effects.

## Results and Discussion

**Fig 2:**
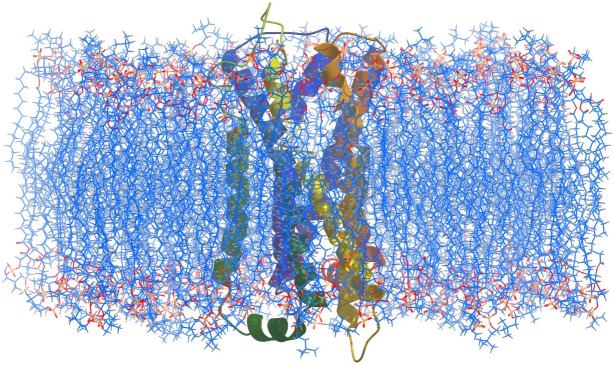
A cross-sectional view of the D3R in membrane.

As discussed above, before embedding the protein-ligand complex in the lipid bilayer, the POPC lipid bilayer was equilibrated for 50 ns. The membrane thickness calculated in last frame of equilibration of POPC (50 ns), using the GridMat-MD, a grid-based membrane analysis,[57] was found to be 34.152 ± 0.11Å (Fig 2). The average cross-sectional area per lipid was also evaluated and was found to be 69.73± 2.5 Å^2^, in agreement with other studies.[61–63]

**Fig 3:**
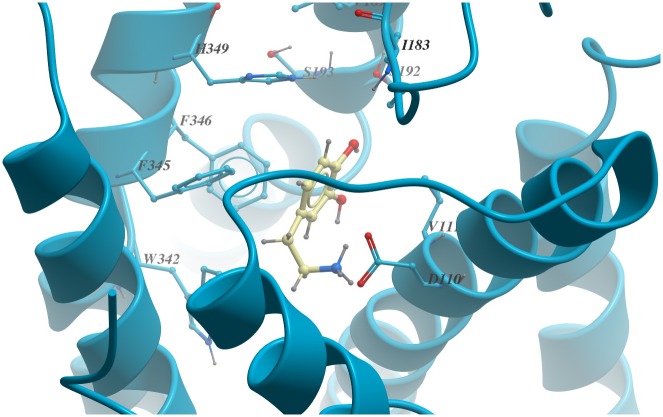
Binding pose of the dopamine in the D3R.

All simulations showed that the dopamine has an almost stable binding pose in the pocket. As shown in Fig 3, the dopamine has had strong interactions with Asp110 and Ser192 and it also has hydrophobic interactions with Val111, Trp342, Val189, Ile183, Phe345, Phe346 and His349.

### A. Application of an oscillating electric field along the z-direction at different frequencies

**Fig 4:**
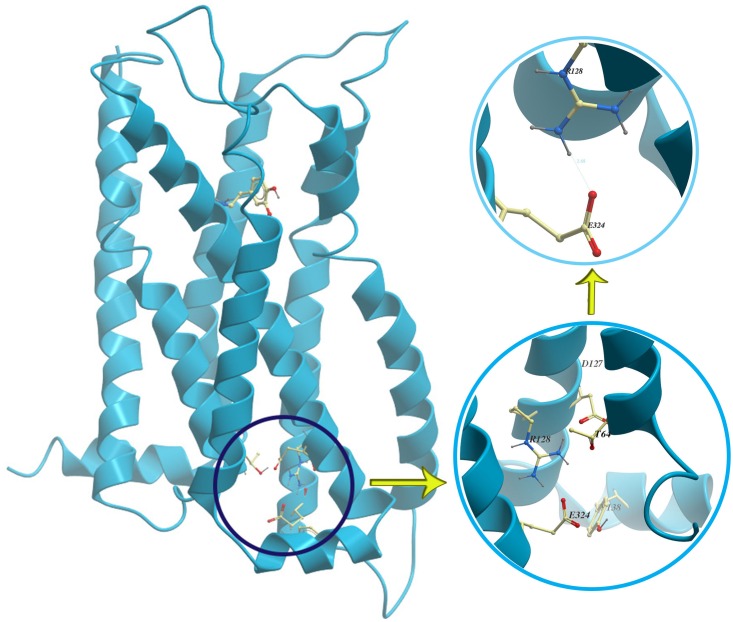
The ionic lock in the D3R.

**Fig 5:**
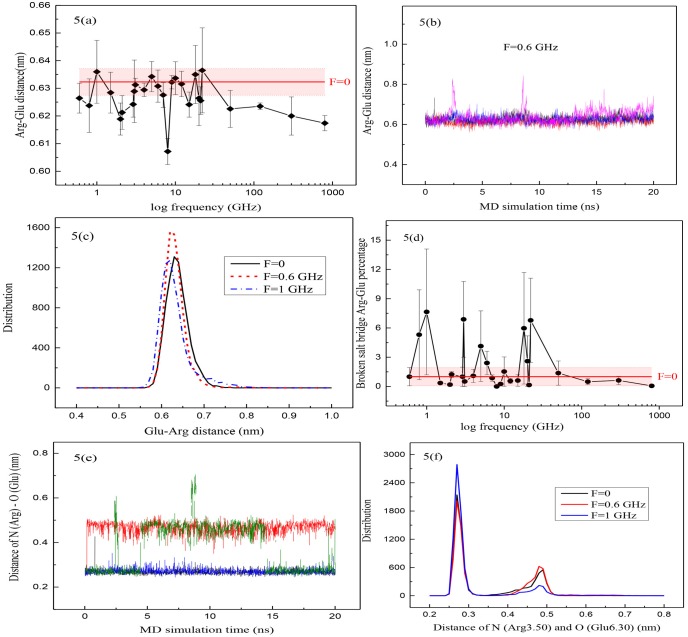
Some plots related to Arg-Glu distance. (a) Arg-Glu distance in different logarithmic frequencies. (Red horizontal-line means no applied electric field and the red shadow is its error bar). (b) Arg128^{3.50}^-Glu324^{6.30}^ distance in 4 independent MD simulations at frequency of 0.6 GHz. (c) Histogram related to Arg128^{3.50}^ and Glu324^{6.30}^ distribution at frequencies 0.6 (red short dash line) and 1 (blue dash dot line) GHz and without applying the oscillating electric field (black solid line). (d) Broken salt bridge parameter corresponds to Arg.Glu at different logarithmic frequencies. (e) Distance between Nitrogen of Arg and Oxygen of Glu in 4 independent MD simulations. (f) Histogram related to N-O distance of Arg.Glu at frequencies 0.6 and 1 GHz and without applying oscillating electric field.

Due to the importance of the ionic lock in activating GPCRs (Fig 4), the distance between the center of mass (CoM) of Arg128^{3.50}^ and Glu324^{6.30}^ was measured at different frequencies (Fig 5(a)). As was mentioned above, the 20 ns simulations at F = 0 and different frequencies were performed four times (Fig 5(b)), so every point on every plot is the average of 4 independent simulations for that quantity (Fig 5(a)). After calculating the distance between CoM of Arg128^{3.50}^ and Glu324^{6.30}^, its histogram was sketched showing the distribution of Arg128^{3.50^} and Glu324^{6.30}^ distance (Fig 5(c)). When the Arg128^{3.50}^ and Glu324^{6.30}^ distance was more than 0.7 Å, the salt bridge between them was considered broken. So we introduced a new parameter which is obtained from the histogram for every MD simulation called the broken salt bridge Arg-Glu percentage. It is a time ratio between the number of states whose distance is more than 0.7 Å and all other states in the histogram. This parameter indicates the probability of breaking the salt bridge. Fig 5(d) shows the broken salt bridge Arg-Glu percentage at different frequencies. We normalized the parameter at all frequencies to F = 0 (no electric field) i.e., when there is no oscillating electric field the parameter = 1. As a result, we can compare the probability of breaking of the salt bridge at different frequencies. As is shown, by considering a range of frequencies, i.e., 0.6, 0.8, 1, 1.5, 2, 2.1, 2.9, 3, 3.1, 4, 5, 6, 7, 8, 9, 10, 12, 15, 18, 20, 21, 22, 50, 120, 300 and 800 GHz along the z-direction, on the logarithmic scale, and considering the error bars in Fig 5(a) and 5(d) and also P-value calculation (S1 Table), in F = 2, 8 and 800 GHz (P-value<0.05), application of oscillating electric field has a significant effect on Arg-Glu distance, but there is no order in changes. The probability for breaking of the salt bridge at frequencies of 0.8, 1, 3, 5, 18 and 22 GHz was higher, but these changes were chaotic and there is no enough evidence in predicting these changes and determining the precise behavior at a special frequency. By considering the diversity of bonds, angles and dihedrals frequency in protein, which is 10^14^−10^9^ Hz, it could be suggested that the fluctuation of different calculated quantities versus frequency and their unpredictability may be due to existence of different collections of bonds, angles and/or dihedral which resonate in each frequency. Each of these collections have different effect on the calculated quantities based on their position and characteristics.

The distance between the atom N of guanidinium group of Arg128^{3.50}^ and the atom O of the side chain carboxylic acid of Glu324^{6.30}^ was also computed (Fig 5(e)). As is shown, in four independent simulations, the distance was changed between two distinct values because of switching of the atom O in Glu324^{6.30}^ with one of the N atoms of guanidinium group in Arg128^{3.50}^. Its histogram (Fig 5(f)) shows apparently a bimodal distribution which indicates two distinct regions with maximum values at 2.7 and 4.9 nm which implies that two the atoms around the average bonding distance have undergone fluctuation. This is due to the delocalization of charge in the guanidinium group, as a result of the conjugation between the double bond and the nitrogen lone pairs in guanidinium group of Arg128^{3.50}^. It is obvious that the behavior of the distance between the N and the O atoms at different frequencies was in accordance with the distance of the center of mass of Arg128^{3.50}^ and Glu324^{6.30}^.

**Fig 6:**
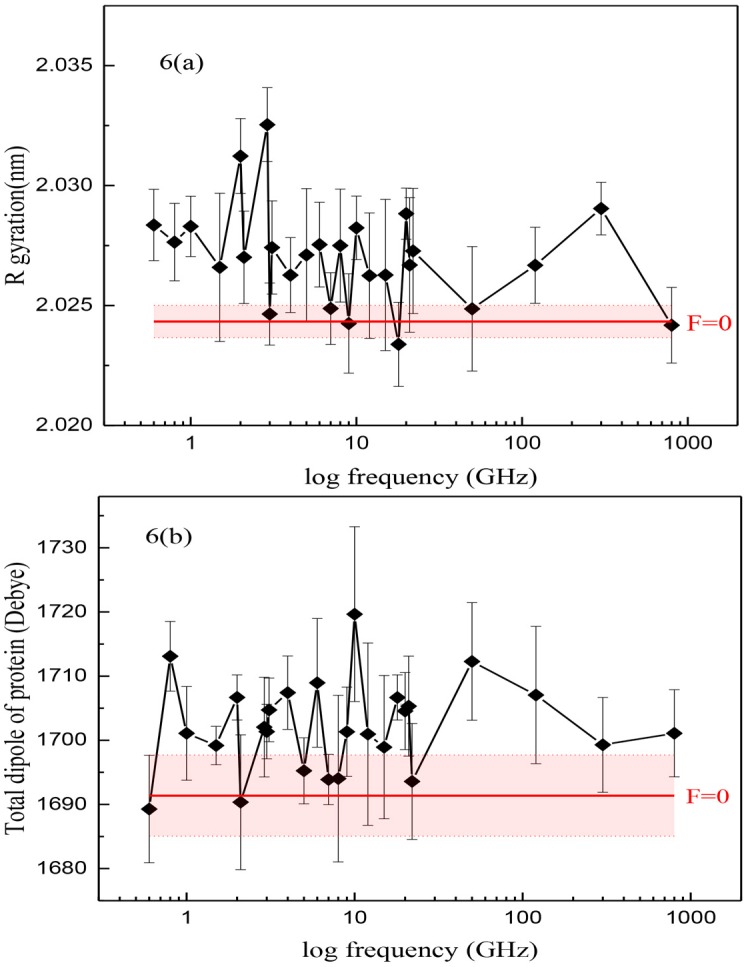
Two quantities of protein at different frequencies. (a) Radius of gyration of the D3R b) Total dipole of the protein at different logarithmic frequencies. (Red horizontal-line means no applied electric field and the red shadow is its error bar).

Other quantities related to the ligand-protein complex such as energy and number of hydrogen bonds between the dopamine and its receptor and their distance, the binding free energy calculated via the LIE method, the radius of gyration of protein, the number of hydrogen bonds of protein and also the number of hydrogen bonds between helix7 and helixes 1, 2, 3, 6 which is important in the activation process as well as the electric dipole moment of protein were computed. Similar behavior was observed, for example, in Fig 6a and 6b the radius of gyration of protein and the total electric dipole moment of the protein at different frequencies are shown, considering the error bars and P-value calculation (S2 Table), at some frequencies such as 0.8, 6 and 10 GHz which have P-value<0.05, applying oscillating electric field has considerable effect on radius of gyration of protein and also total dipole of protein but there is no predictable change at special frequencies when frequency of the oscillating electric field increases in the z-direction.

To have a deeper insight into the changes observed at different frequencies, we calculated the correlation coefficients between various quantities of the system during simulation (Table 1) and for the probability of accuracy of correlation coefficients, t-test calculation has been done (α = 0.05, n = 27). For example, the correlation coefficient showed that increasing the logarithmic frequency may make the distance between Arg128{3.50}and Glu324{6.30} smaller (the coefficient value was about -0.25 with 90% significance). This may mean that by increasing the frequency, the ionic lock may be stabilized. It also shows that with an increase in the logarithmic frequency, the distance between the ligand and the protein increases; it may imply that by increasing the electric field frequency, the interaction between the dopamine and the D3R becomes weaker (Correlation coefficient = 0.45 with 95% significance). So by a slightly significant claim, there is a weak correlation (-0.22) between the distances of the center of mass of Arg128{3.50}-Glu324{6.30} and the energy between the ligand and the protein, so that by increasing the energy between the ligand and the protein, the distance between Arg128{3.50} and Glu324{6.30} may decrease. Thus, if the contact between the dopamine and the D3R increases, the salt bridge may weaken. This can affirm the role of breaking of salt bridge within activation process in the D3R. Consequently, it can be concluded that applying a high frequency oscillating electric field may stabilize the ionic lock and decreases the interaction between the dopamine and the D3R.

**Table 1:** Correlation coefficient between different variables.

Variables	1	2	3	4	5	6	7	8	9	10	11	12	13	14
1. Frequency	1													
2. logF	0.72	1												
3. Arg-Glu Distance	**-0.36	**-0.25	1											
4. Broken Bond Arg-Glu%	**-0.41	**-0.28	0.99	1										
5. Energy ionic lock	0.02	0.05	0.07	0.08	1									
6 Energy Ligand-Protein	***0.52	**0.29	**-0.22	**-0.22	-0.05	1								
7. Ligand-Protein Distance	**0.5	**0.45	0.02	-0.02	0.02	0.02	1							
8. HB Ligand-Protein	-0.24	-0.01	0.07	0.05	0.06	***-0.81	**-0.41	1						
9. Binding Free Energy	0.25	-0.003	**-0.17	**-0.21	0.17	0.19	**0.33	-0.19	1					
10. Energy Protein	-0.16	-0.22	0.03	0.02	0.16	0.13	0.07	-0.03	0.12	1				
11. Radius Gyration Protein	-0.2	-0.32	**-0.22	**-0.23	-0.01	-0.11	**-0.26	0.24	0.02	***0.53	1			
12. HB Protein-Protein	-0.01	-0.13	**0.35	**0.35	0.01	-0.02	-0.08	-0.08	-0.18	***-0.61	-0.38	1		
13. Dipole	-0.01	0.13	0.09	0.06	**-0.25	0.17	0.13	-0.14	-0.18	-0.17	0.09	0.06	1	
14. Variance of Dipole	***-0.68	***-0.57	**0.42	**0.47	-0.08	-0.23	-0.23	0.09	-0.15	0.03	0.01	**0.3	-0.04	1

Data marked with ** and *** (moderate and strong correlation) are considered more important.

Functional connectivity shows that the variance of the electric dipole moment of the protein decreases by increasing the frequency, so at higher frequencies although the electric dipole moment of the D3R does not change significantly but its variation will, however, decrease. When the distance of the center of mass of the salt bridge increases, the variance of the electric moment of the D3R would increase. It can be concluded that under applying an external oscillating electric field, when some events lead to an increase in the variance of the protein’s electric moment, it can force the receptor, via breaking of ionic lock, to be activated.

It is obvious that by increasing the distance between the dopamine and the D3R, the binding free energy will increase and the number of hydrogen bonds between the ligand and the receptor will decrease. This is confirmed by correlation coefficients. Under an external electric field, by increasing the energy of the protein, the radius of gyration will increase and the number of internal hydrogen bonds will decrease, which is rational.

There is also some almost weak correlation between some quantities; for example, by increasing the variance of the electric moment of the D3R, the total internal hydrogen bonds of the protein will increase. Furthermore, by increasing the energy of the ionic lock, the total electric dipole moment of the protein decreases. According to what was mentioned above, a decrease in the total electric moment of the protein and the simultaneous increase in the variance of the electric moment may raise the probability of the breaking of the ionic lock.

### B. Application of high amplitude field in the z-direction

To investigate further the effect of an oscillating electric field and considering the very limited time of molecular simulations, the amplitude of the oscillating electric field was increased by 10 folds. Because of the low error bar at frequency of 6 GHz and the importance of this frequency in the communication technology, a high amplitude field was applied at this frequency in the z-direction.

Comparing the results pertinent to 0.0919 and 0.919 V/nm amplitudes shows that there are some changes in some quantities when the field intensity is increased; for instance, by considering the error bars, the energy of the ionic lock and the number of internal hydrogen bonds of the protein and the radius of gyration as well as the variance of the electric dipole moment of the protein are decreased (S3 Table). These changes are not, however, significant and applying an oscillating electric field in the z-direction cannot have a significant effect on the D3R as discussed above for different frequencies. For example, the distance between Arg128^{3.50}^ and Glu324^{6.30}^ at the frequency of 6 GHz was found to be 0.626 Å ± 0.0054 which was changed to 0.624 Å ± 0.0007 when a high amplitude electric field was applied. Considering the simulation trajectory and its results, it can be concluded that the D3R in the z-direction, which is in the direction of α helices, is so stable that even applying an intense oscillating electric field cannot produce any considerable effect.

### C. Application of oscillating field in other directions

To gain a better insight into the effect of the application of an oscillating electric field on the dopamine-D3R complex, role of field’s direction was also investigated. Oscillating electric fields at the frequency of 6 GHz were applied in the Y-Z plane with angles of 45 and 30 degrees and also along the X-Y plane.

**Fig 7:**
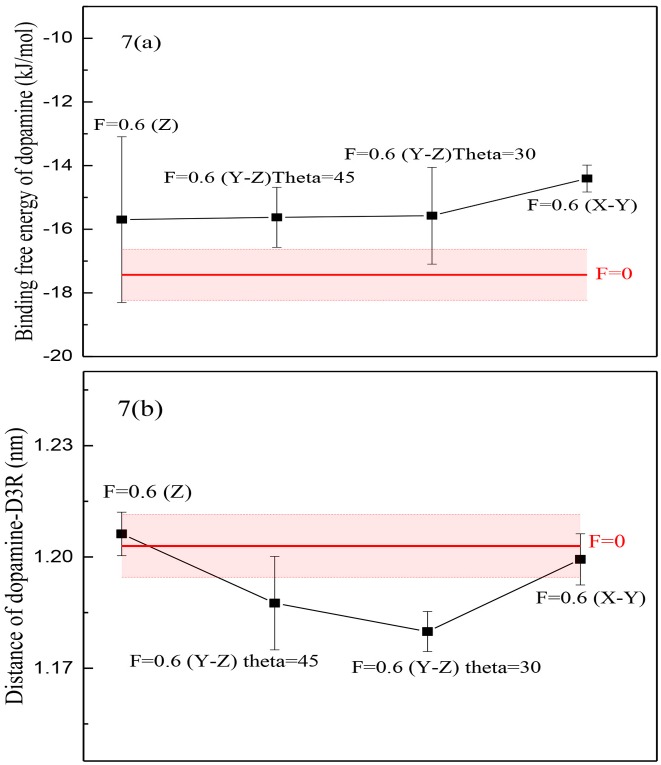
Two parameters in different directions of oscillating electric field. (a) Binding free energy of dopamine, (b) Distance between dopamine and D3R by applying an external oscillating electric field in different directions ((Z): Electric field along the z-direction, (Y-Z) θ = 30 and 45: Electric field in the Y-Z plane with angles of 30 and 45 respectively, (X-Y): Electric field along the X-Y plane, Red horizontal-line means no applied electric field and the red shadow is its error bar).

It is interesting that there was some considerable effect by changing the orientation of the electric field. As shown in Fig 7 and calculated P-values of binding free energy and distance between dopamine and D3R (S4 Table), at the same amplitude of the electric field, binding free energy of dopamine under an alternative electric field in the Y-Z plane (θ = 45 and 30) was less than its value when no electric field was applied, and both of these were less than the electric field when applied in the X-Y plane (θ = 30). It also seemed that applying an oscillating electric field in the Y-Z plane has an effect on the distance and energy between the dopamine and its D3R; this can be due to the relative orientation of the ligand and the receptor. Consequently, it can be concluded that the orientation of the oscillating electric field is important, which can be open to further investigation.

Applying an oscillating electric field along in the X-Y plane has an effect on the receptor; for instance, the distance of Arg-Glu decreased when external electric field was applied in Y-Z plane (θ = 30) and energy of the receptor will also increase while the total number of internal hydrogen bonds of the protein will decrease (S1 Fig).

## Conclusions

Applying an external electric field, at different frequencies, in the logarithmic scale along the z-direction on the dopamine-D3R complex, at some frequencies has a significant effect on salt bridge interaction and the structural observables, although it does not have any systematic or predictable effect on the dynamical and physical property of the protein. Correlation coefficient between different variables showed that increasing the frequency increased the distance between dopamine and D3R. So increasing the frequency can weaken the interaction between the ligand and the receptor and may stabilize the ionic lock. This can be an affirmation for the role of the salt bridge breaking within the activation process in the D3R.With almost weak correlation, it can be concluded that decreasing the total electric moment of the protein and simultaneously increasing the variance of the electric moment may raise the probability of breaking the ionic lock. Applying an intense oscillating electric field along the z-direction which is along the axis of stable α helices cannot have a very significant effect on dopamine-D3R complex while, on the other hand, changing the orientation of the oscillating electric field may be important and can have significant structural effect.

## Supporting Information

S1 Fig Some parameters of dopamine-D3R complex in different directions of oscillating electric field. (a) Energy of ionic lock, (b) Arg-Glu distance, (c) Energy of D3R, (d) Number of internal hydrogen bond of D3R, (e) Binding free energy of dopamine by applying an external oscillating electric field in different directions ((Z): Electric field along the z-direction, (Y-Z) θ = 30 and 45: Electric field in the Y-Z plane with angles of 30 and 45 respectively, (X-Y): Electric field along the X-Y plane and Red horizontal-line means no applied electric field and the red shadow is its error bar).
(TIF)

S1 Table P-Values of Glu-Arg distance and Broken salt bridge Arg-Glu percentage in different frequencies. P-Value<0.05 means that this frequency has a significant effect on Glu-Arg distance or Broken salt bridge Arg-Glu percentage (Null hypothesis is rejected).
(PDF)

S2 Table P-Values of radius of gyration and total dipole of protein in different frequencies. P-Value<0.05 means that this frequency has a significant effect on radius of gyration or total dipole of protein (Null hypothesis is rejected).
(PDF)

S3 Table Different quantities by applying high amplitude of oscillating electric field. (PDF)

S4 Table P-Values of different quantities of dopamine-D3R complex in different direction of external oscillating electric field. P-Value<0.05 means that applying oscillation field in that direction has a significant effect on that quantity (Null hypothesis is rejected).
(PDF)
