# Supplementary figures and images for: Structural and Functional Effect of an Oscillating Electric Field on the Dopamine-D3 Receptor: A Molecular Dynamics Simulation Study
Source: PLoS One. 2016 Nov 10;11(11):e0166412. doi: 10.1371/journal.pone.0166412 (PMC5104473; doi:10.1371/journal.pone.0166412)

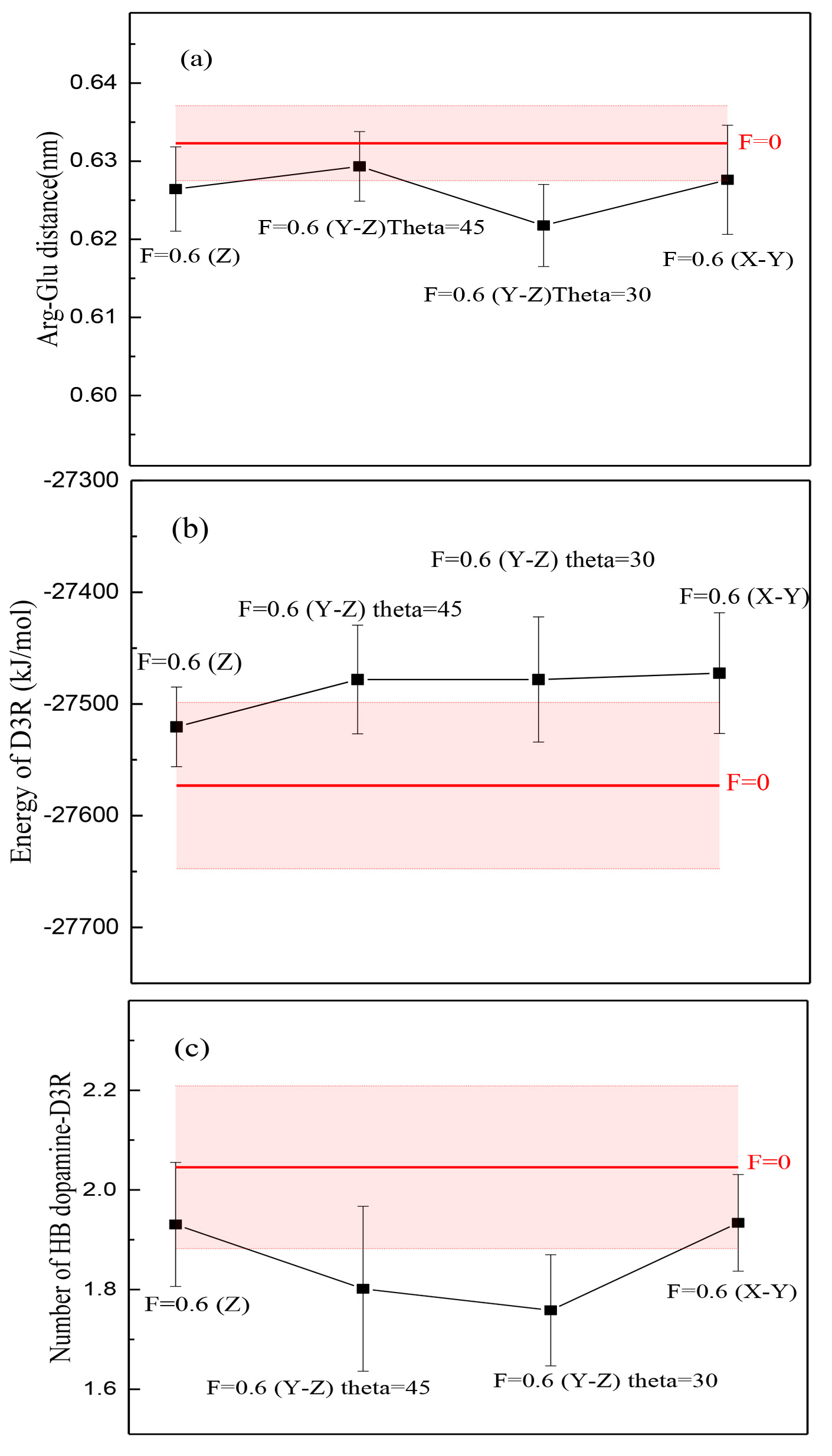

Supplement: S1 Fig — (a) Energy of ionic lock, (b) Arg-Glu distance, (c) Energy of D3R, (d) Number of internal hydrogen bond of D3R, (e) Binding free energy of dopamine by applying an external oscillating electric field in different directions ((Z): Electric field along the z-direction, (Y-Z) θ = 30 and 45: Electric field in the Y-Z plane with angles of 30 and 45 respectively, (X-Y): Electric field along the X-Y plane and Red horizontal-line means no applied electric field and the red shadow is its error bar). (TIF) [file pone.0166412.s001.tif]
