# Supplementary material for: Structural and Functional Effect of an Oscillating Electric Field on the Dopamine-D3 Receptor: A Molecular Dynamics Simulation Study
Source: PLoS One. 2016 Nov 10;11(11):e0166412. doi: 10.1371/journal.pone.0166412 (PMC5104473; doi:10.1371/journal.pone.0166412)
Supplement: S1 Table — P-Value<0.05 means that this frequency has a significant effect on Glu-Arg distance or Broken salt bridge Arg-Glu percentage (Null hypothesis is rejected). (PDF) [file pone.0166412.s002.pdf]

S1 Table. P-Values of Glu-Arg distance and Broken salt bridge Arg-Glu percentage in different frequencies.

Frequency (GHz)	P-Value Glu-Arg distance	P-Value Broken salt bridge Glu-Arg percentage
0.6	0.24	0.5
0.8	0.15	0.003
1	0.33	0.00002
1.5	0.32	0.35
2	0.05	0.31
2.1	0.09	0.44
2.9	0.16	0.5
3	0.34	0.0001
3.1	0.45	0.38
4	0.36	0.48
5	0.41	0.03
6	0.43	0.19
7	0.28	0.47
8	0.001	0.27
9	0.5	0.32
10	0.44	0.37
12	0.46	0.39
15	0.16	0.4
18	0.37	0.001
20	0.24	0.16
21	0.21	0.3
22	0.31	0.0002
50	0.12	0.41
120	0.15	0.38
300	0.07	0.41
800	0.04	0.28

P-Value<0.05 means that this frequency has a significant effect on Glu-Arg distance or Broken salt bridge Arg-Glu percentage (Null hypothesis is rejected).
