# Supplementary material for: Structural and Functional Effect of an Oscillating Electric Field on the Dopamine-D3 Receptor: A Molecular Dynamics Simulation Study
Source: PLoS One. 2016 Nov 10;11(11):e0166412. doi: 10.1371/journal.pone.0166412 (PMC5104473; doi:10.1371/journal.pone.0166412)
Supplement: S2 Table — P-Value<0.05 means that this frequency has a significant effect on radius of gyration or total dipole of protein (Null hypothesis is rejected). (PDF) [file pone.0166412.s003.pdf]

S2 Table. P-Values of radius of gyration and total dipole of protein in different frequencies.

Frequency (GHz)	P-Value Radius of Gyration	P-Value Total Dipole of Protein
0.6	0.0002	0.42
0.8	0.002	0.02
1	0.0003	0.19
1.5	0.02	0.24
2	0	0.08
2.1	0.01	0.46
2.9	0	0.16
3	0.39	0.18
3.1	0.004	0.11
4	0.05	0.07
5	0.01	0.36
6	0.003	0.05
7	0.32	0.41
8	0.003	0.4
9	0.47	0.18
10	0	0.0005
12	0.05	0.19
15	0.05	0.25
18	0.21	0.08
20	0.00005	0.11
21	0.02	0.1
22	0.006	0.42
50	0.32	0.03
120	0.02	0.08
300	0	0.23
800	0.45	0.19

P-Value<0.05 means that this frequency has a significant effect on radius of gyration or total dipole of protein (Null hypothesis is rejected).
