# Supplementary material for: Structural and Functional Effect of an Oscillating Electric Field on the Dopamine-D3 Receptor: A Molecular Dynamics Simulation Study
Source: PLoS One. 2016 Nov 10;11(11):e0166412. doi: 10.1371/journal.pone.0166412 (PMC5104473; doi:10.1371/journal.pone.0166412)
Supplement: S3 Table — (PDF) [file pone.0166412.s004.pdf]

S3 Table. Different quantities by applying high amplitude of oscillating electric field.

	Amplitude=0.0919 V/nm	Error bar	Amplitude=0.919 V/nm	Error bar
Energy of ionic lock (kJ/mol)	-230.40	±17.97	-262.39	±7.74
Number of internal HB of protein	214.57	±0.64	212.33	±0.16
Radius of gyration (nm)	2.028	±0.0014	2.025	±0.0010
Variance of dipole of protein	6461.93	±937.43	4632.23	±295.48
Arg-Glu distance (nm)	0.626	±0.0054	0.624	±0.0007
Binding free energy of dopamine (kJ/mol)	-15.69	±2.60	-14.56	±1.25
