# Supplementary material for: Structural and Functional Effect of an Oscillating Electric Field on the Dopamine-D3 Receptor: A Molecular Dynamics Simulation Study
Source: PLoS One. 2016 Nov 10;11(11):e0166412. doi: 10.1371/journal.pone.0166412 (PMC5104473; doi:10.1371/journal.pone.0166412)
Supplement: S4 Table — P-Value<0.05 means that applying oscillation field in that direction has a significant effect on that quantity (Null hypothesis is rejected). (PDF) [file pone.0166412.s005.pdf]

S4 Table. P-Values of different quantities of dopamine-D3R complex in different direction of external oscillating electric field.

Different direction of Electric Field at F=0.6 GHz	P-value Binding free energy of dopamine	P-value Distance between dopamine and D3R
Z	0.02	0.41
Y-Z θ=45	0.01	0.15
Y-Z θ=30	0.01	0.05
X-Y	0.0001	0.4

P-Value<0.05 means that applying oscillation field in that direction has a significant effect on that quantity (Null hypothesis is rejected).
